# Design of a randomized controlled trial on the effect on return to work with coaching plus light therapy and pulsed electromagnetic field therapy for workers with work-related chronic stress

**DOI:** 10.1186/s12889-016-3276-6

**Published:** 2016-07-19

**Authors:** Antonius M. C. Schoutens, Monique H. W. Frings-Dresen, Judith K. Sluiter

**Affiliations:** Academic Medical Center, Department: Coronel Institute of Occupational Health, Amsterdam Public Health Research Institute, Amsterdam, The Netherlands; FluxPlus BV, Tilburg, The Netherlands; Coronel Institute of Occupational Health, Academic Medical Center, Meibergdreef 9, 1105 AZ, Amsterdam, The Netherlands

**Keywords:** Work-related chronic stress, Burnout, Light therapy, Pulsed electromagnetic field therapy (PEMF), Work participation, Stress, Fatigue, Quality of life

## Abstract

**Background:**

Work-related chronic stress is a common problem among workers. The core complaint is that the employee feels exhausted, which has an effect on the well-being and functioning of the employee, and an impact on the employer and society. The employee’s absence is costly due to lost productivity and medical expenses. The usual form of care for work-related chronic stress is coaching, using a cognitive-behavioural approach whose primary aim is to reduce symptoms and improve functioning. Light therapy and pulsed electromagnetic field therapy are used for the treatment of several mental and physical disorders. The objective of this study is to determine whether coaching combined with light therapy plus pulsed electromagnetic field therapy is an effective treatment for reducing absenteeism, fatigue and stress, and improving quality of life compared to coaching alone.

**Methods/design:**

The randomized placebo-controlled trial consists of three arms. The population consists of 90 participants with work-related chronic stress complaints. The research groups are: (i) intervention group; (ii) placebo group; and (iii) control group. Participants in the intervention group will be treated with light therapy/pulsed electromagnetic field therapy for 12 weeks, twice a week for 40 min, and coaching (once a fortnight for 50 min). The placebo group receives the same treatment but with the light and pulsed electromagnetic field switched to placebo settings. The control group receives only coaching for 12 weeks, a course of six sessions, once a fortnight for 50 min. The primary outcome is the level of return to work. Secondary outcomes are fatigue, stress and quality of life. Outcomes will be measured at baseline, 6 weeks, 12 and 24 weeks after start of treatment.

**Discussion:**

This study will provide information about the effectiveness of coaching and light therapy plus pulsed electromagnetic field therapy on return to work, and secondly on fatigue, stress and quality of life in people with work-related chronic stress.

**Trial registration:**

NTR4794, registration date 18-sept-2014

## Background

Work-related chronic stress (burnout) is the psychological concept of having high work demands and the feeling of being exhausted, having no energy or motivation for performing activities related to work, and will lead in most cases to partial failure or even to complete breakdown [1]. The term burnout was used for the first time in the early 1970s by the American psychotherapists Herbert Freudenberger and Christina Maslach [2], with their theory becoming dominant in the field. According to Maslach, burnout consists of three more or less related phenomena: (i) exhaustion (a feeling of extreme tiredness); (ii) cynicism (distance to work, or to the people with whom one works); and (iii) reduced personal accomplishment (the feeling of being less able to carry out work, including reduced work-related self-confidence). People who have a burnout have little energy to start new activities, feel exhausted and are no longer able to carry out work. The incidence of severe strain in work-related chronic stress was estimated at 307 per 100,000 workers in the Netherlands in 2011 [3]. Work-related chronic stress is an increasing problem for workers and employers and the costs to society are substantial [4].

The regular approach for workers with work-related chronic stress complaints with sickness absence is mental coaching, based on cognitive-behavioural insights [5]. In the coaching sessions, factors are identified that influence the occurrence of the symptoms. These factors are then addressed to alleviate the symptoms. A treatment consists of several stages. In the initial phase of therapy, reduction of symptoms and global recovery are the primary targets. The condition improves and physical symptoms are reduced. The subsequent phases consist of teaching skills and researching dysfunctional thoughts that perpetuate the symptoms. These last two phases can be reversed in sequence.

Light therapy appears to be effective in reducing fatigue in burnout [6], while the treatment with a weak magnetic field (Pulsed Electro Magnetic Field, PEMF) has been used with some success in treatment-resistant depression [7] and pain [8]. The combination of light therapy and electromagnetic field therapy in the treatment platform Xentix [9] is a promising additional treatment. Light therapy is known to stabilize the sleep/wake rhythm and this may result in increased energy and reduced fatigue. Pulsed Electromagnetic Field therapy stimulates the metabolism, creating a better balance between the cell and the intercellular space [10]. Hormones and neurotransmitters move from one cell type to another and carry chemical “messages” that modulate the metabolic responses of tissues to the environment. Interaction with these signalling systems is a potential mechanism by which very low-energy electromagnetic fields might produce metabolic responses in the body. Hormone and neurotransmitter receptors are specialized protein molecules that use a variety of biochemical activities to pass chemical signals from the outside of a cell across the plasma membrane to the interior of the cell. Since many low-energy electromagnetic fields have too little energy to directly traverse the membrane, it is possible that they may modify the existing signal transduction processes in cell membranes, thus producing both transduction and biochemical amplification of the effects of the field itself [11]. The current state of knowledge of the physiological mechanisms of PEMF remains limited however.

Both light therapy for burnout [6] and magnetic field therapy in fibromyalgia [12] show a positive effect on emotional exhaustion, one of the main symptoms of burnout. Light therapy also has a positive effect on fatigue and quality of life in patients with SAD (Seasonal Affective Disorder SAD) and winter blues (Sub-Syndromal Seasonal Affective Disorder S-SAD) [13, 14]. In Multiple Sclerosis (MS), a positive effect of light therapy has been seen on fatigue [15, 16] and of magnetic field therapy on quality of life [17]. It is expected that the intervention with coaching and light therapy plus magnetic field therapy will lead to a faster return to work and increased reduction of stress levels and fatigue. This could lead to an improvement in the degree of work participation and of the quality of life. Consequently, medical consumption is thought to be reduced.

The aim of this study is to establish whether an approach incorporating coaching supplemented by light therapy plus pulsed electromagnetic field therapy in people with work-related chronic stress symptoms contributes to earlier return to work compared to guidance with coaching only. A secondary aim is to assess the effect on the reduction of fatigue, chronic stress and quality of life.

## Methods

### Study design

The study is a three-armed randomized placebo-controlled trial (RCT) with patients with work-related chronic stress (*n* = 90). Subjects with the aforementioned problems are treated in three groups: (i) group 1 receives light therapy/electromagnetic field therapy and coaching (CoLMF group); (ii) group 2 receives the same treatment conditions but the light therapy/electromagnetic field therapy is not activated (CoPL group); and (iii) group 3 will receive coaching only (Co group).

### Setting

**Fig. 1:**
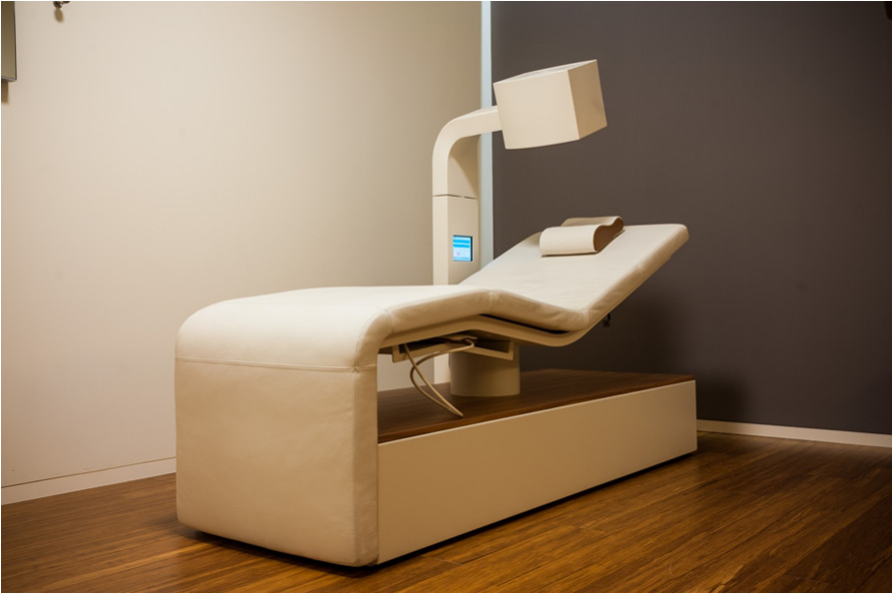
Xentix treatment platform

The study will be performed in a treatment centre in Eindhoven, the Netherlands that has been built and equipped specifically for this study. An advanced light therapy/magnetic therapy treatment platform was developed in Belgium, which, in combination with coaching, might alleviate the symptoms in people suffering from work-related chronic stress. The centre offers five treatment rooms where the treatment equipment is set up, and two rooms for coaching. The staff consists of researchers, coaches and trained assistants. The device used in the study is a reclining chair with a lengthened seat forming a leg rest (see Fig. 1). It forms a comfortable treatment platform with a light therapy unit mounted above the participant’s head. The electromagnetic coils are situated in the pillow and bed. During the treatment, the participants lie on the bed. Their eyes are closed so that the light reaches the retina through the eyelids. In the case of high photosensitivity of the participant, dark goggles can be worn.

### Study population

The population consists of employees (18–65 years of age) from the south of the Netherlands (Eindhoven region) with work-related chronic stress complaints with at least 50 % absenteeism.

### Recruitment of the participants

Participants are recruited through social media, newspapers, general practitioners and through an occupational health service. Prior to the recruitment of participants, all staff will be informed.

### Inclusion criteria

- Employees with work-related chronic symptoms aged between 18 and 65 years with at least 50 % sickness absenteeism
- Diagnostic criteria for neurasthenia (symptoms 0–6 months)
- Speaking the Dutch language

### Exclusion criteria

- Pregnancy
- Serious somatic problems such as diabetes or epilepsy
- Serious ocular diseases
- Confusion or severe gloom
- Use of psychotropic drugs other than selective serotonin reuptake inhibitors SSRIs
- Pacemaker/neuro-stimulators

### Informed consent and randomization

**Fig. 2:**
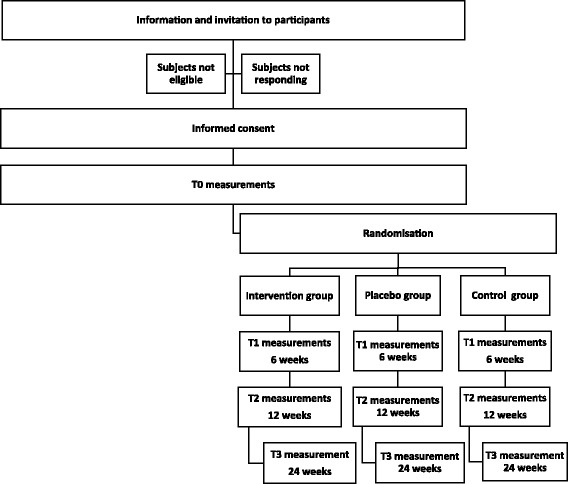
Study design

Randomization (Fig. 2) will take place once the participant meets the inclusion criteria, has read the written information about the study and has given written informed consent after approval and after the baseline measurements. Preliminary numbers are assigned to the three groups of equal size in the study. Participants receive a random number from a research assistant who is not involved in the actual measurements. These numbers are printed on the measuring instruments. Participants are given a random number on the basis of entry and assigned to one of the three groups.

### Blinding

The RCT is single-blinded in the sense that none of the outcome assessors are aware of the group assignment of the subjects. Furthermore, because of the use of a placebo-controlled group, in two out of the three groups the participants are double-blinded.

### Intervention

#### Coaching

The regular guidance during mental coaching sessions of people with work-related chronic stress is a cognitive-behavioural approach [18, 19]. A standard guidance protocol is used by certified coaches (BSc, MSc) who were originally trained as work and organization psychologists, clinical therapists or mental health counsellors. In addition, they have all been additionally trained as a mental coach. The guidance helps in identifying and solving the problems the employee has. During coaching, the three stages of recovery are defined: (i) acceptation and relaxation; (ii) increasing employability; and (iii) support to return to work. The guidance consists of a number of regular and elective modules. The key components of the modules are: (i) to reduce symptoms; (ii) solution focused therapy; and (iii) relapse prevention. Additionally, time management, organisation-focused interventions, conflict management, psycho-education and ways to influence fatigue and restore the balance between capacity and payload are mainly addressed. Participants are taught to set new personal goals and to achieve them.

#### Light therapy

Electromagnetic energy consists of a combination of coherent and incoherent light. The coherent light is generated through multiple high-performance LEDs (light emitting diodes) with wavelengths of 470 nm (blue), 525 nm (green) and 570 nm LED (yellow). The non-coherent light is generated by means of an optically-centred halogen lamp with a light output of +/− 9000 lumens. The maximum illuminance at the eye is +/− 6000 l×. The light is directed by means of facetted and coated reflectors and then linearly polarized. Ultra Violet (UV) and Infra-Red (IR) wavelengths are filtered out of the spectrum. An important role is played by a specific type of ganglion cell in the retina involving intrinsic light-sensitive retinal ganglion cells Ip-RGC (intrinsic photo-sensitive retinal ganglion cells). Like other retinal ganglion cells, these are concerned with processing information collected by the rods and cones. One of the differences with the other ganglion cells is that Ip-RGCs also are directly sensitive to light. They contain the photosynthetic pigment melanopsin, which is most sensitive to blue-green light (absorption maximum near 480 nm). Another difference relates to anatomy. Ip-RGCs appear at various locations in the retina, and they project to several specific areas in the brain. One target of the projections of the Ip-RGCs is the Suprachiasmatic Nucleus (SCN), also known as the biological clock.

#### Pulsed electro Magnetic field therapy PEMF

The weak magnetic fields are generated in the apparatus using low voltage in multiple coils that are integrated and spread over the sofa and in the pillow set. The magnetic fields are wide-band and the amplitude of the magnetic fields is almost constant across the whole of the frequency band used. The specific frequency band is in the ELF (extremely low frequency, 0-30Hz) and the ULF (ultra-low frequency, 30–30,000Hz) ranges. The maximum magnetic flux density B = 0.3 Wb/m^2^ = 0.3 T. The principle of operation is hypothesized to be due to a better cell metabolism whereby ions are transferred more quickly and easily between the cell and the intercellular space.

#### Frequency and duration of the interventions

Participants will be treated for 12 weeks, twice a week for 40 min on the treatment platform with light therapy/magnetic field therapy, with coaching being given 50 min every fortnight during a 12-week period (CoLMF group 1). The placebo group receives the same treatment condition but the light and magnetic field are switched off (CoPL group 2). The control group receives coaching only (Co group 3), also 50 min once a fortnight during a 12-week period.

#### Control interventions

The participants allocated to the placebo group (2) will undergo treatment at the treatment platform but with the magnetic field switched off. Only a non-effective, small dose of coherent/incoherent light > 5 lx is used to give the impression that the apparatus is working. The participants allocated to the coaching group (3) receive regular guidance in coaching sessions given by a certified coach.

### Outcomes

#### Primary outcome

The primary outcome variable is the percentage return to work: the number of contract hours that participants work at the end of the study compared to the number of contract hours worked in the week prior to the study (T0). The number of hours worked will be measured at T0 (start), T2 (after 12 weeks) and T3 (after 24 weeks).

### Secondary outcomes

#### Fatigue

Fatigue will be assessed using two scales. The first scale used is for emotional exhaustion - the UBOS G(eneral) [20]. The Utrecht Burnout Scale UBOS is the Dutch version of the Maslach Burnout Inventory. This study uses only the emotional exhaustion subscale.

The second variable is the Need for Recovery after work scale of the Dutch Experience and Evaluation of Work Questionnaire (Dutch: VBBA, Meijman and Van Veldhoven, 1994) [21]. The scale measures work-related fatigue as the extent to which employees experience problems in recovery efforts from work. The Need for Recovery after work scale score is calculated by adding the individual’s scores on the eleven (recoded) items. This scale score is transformed into a scale ranging from 0 to 100. Higher scores indicate a higher degree of need for recovery after work.

#### Stress

Stress will be measured using the distress subscale of the Four-Dimensional Symptoms Questionnaire (4DSQ) [22]. The 16-item questionnaire uses a 5-point response scale (0 = *no*, 4 = *very often*) and has a Cronbach’s alpha of 0.90.

The accumulation of the stress level of an individual is measured by the stress hormone cortisol in hair. Hair cortisol reflects the cumulative responses over time of cortisol in the body [23]. Using measurements of head hair makes it possible to determine accurately the level of experienced stress that has accumulated over a long period. Hair strands in the posterior vertex region are cut as close as possible to the scalp using iron scissors. Cortisol concentrations will be determined from a 3-cm hair segment. Based on an average hair growth rate of 1 cm per month [24], each hair segment reflects hair grown over approximately 3 months. In this study, cortisol concentrations in the last month are determined at baseline (T0) and after 12 weeks (T2).

#### Quality of life

The Dutch version of the SF36 questionnaire [25] is used to measure the quality of life. This study assesses three dimensions: (i) vitality; (ii) role limitations due to personal or emotional well-being; and (iii) social functioning. For each variable item, scores are coded, summed, and transformed to a scale from 0 (worst possible health state measured by the questionnaire) to 100 (best possible health state measured by the questionnaire).

### Other study parameters

#### Socio-demographic variables

At baseline, socio-demographic data such as age, gender and job title will be collected by the research assistants by means of an interview.

#### Statistical analysis

Data will be analysed using the statistical package IBM SPSS Statistics 22. The baseline data and the data from T1, T2 and T3 will be analysed and stored in a databank. The longitudinal analysis will be used to examine differences between the three arms with regard to improvement of the primary outcome (return to work) and the secondary outcomes of stress, fatigue and quality of life.

#### Sample size

The sample size is based on a medium effect size on the primary outcome of the intervention, return to work. Changes in the outcome measures are measured across the three groups and four measuring points by comparing them at the start of the study (T0) with the outcome measures after the end of the measurement period (T3). In order to determine the differences within and between the four measurement points, a multilevel regression analysis is conducted. For a power of 0.80 with an effect size of f = 0.3, the number of participants required is *N* = 79, with *N* = 90 being a desirable starting figure.

#### Ethical considerations

This study has been approved by the Medical Ethics Assessment Committee of the Academic Medical Center (University of Amsterdam). There are no risks associated with participating in the study. The light intensity of 6000 l× is low compared with daylight (0,1 l× – 100 klx) and the spectrum has no Ultra Violet (UV) or Infra-Red (IR) wavelengths. The weak electromagnetic fields are approximately 300 mT. Confidentiality is guaranteed during the course of the study for the participants of all study arms, as no information about the screening or the interventions will be provided to others, such as employers. Furthermore, the study participants of all study arms retain unrestricted access to other care as usual if requested.

## Discussion

This paper describes a protocol for a three-arm RCT consisting of an intervention in which workers with work-related chronic stress are treated with a combination of coaching and light therapy plus.

Pulsed Electromagnetic Field therapy, PEMF. Typically, work-related chronic stress is understood to be a form of emotional exhaustion and fatigue that is not recovered from within 6 months. Both work-related stressors and personal characteristics play a role in the development of work-related chronic stress. There are substantial limitations in professional and/or social functioning. In the Netherlands this disorder accounts for an average of 185 days of absenteeism per person/year and is burdensome and costly for both the employee and the employer.

There are few technical interventions available for work-related chronic stress. One of the few non-technical interventions is the cognitive-behavioural approach, often supplemented with sedatives. Despite the effectiveness of this guidance, it takes a relatively long period of time before workers recover. Shortening the duration of the reintegration time offers advantages for employees, employers and society in general.

The combination of light therapy and pulsed electromagnetic field therapy is unique and combines two promising treatments. The treatment platform used in the study is easy to use, safe and comfortable. There are no known side effects. The method can be applied quickly and has little effect on the daily life of the client. Unlike coaching, the treatment platform can be used anywhere. In addition, it requires no deployment of personnel and it can be used at any time of the day. Most people appreciate lying on the treatment platform. It relaxes them and they experience rest, and no sedatives are needed. Often the participant falls asleep. If proven effective, the treatment can be applied as a new therapeutic tool for people with work-related chronic stress. The platform can be used by coaches, occupational physicians and other health professionals and therapists in daily practice.

Because the participants in this study will be from a heterogeneous sample of workers (recruited through social media, newspapers, general practitioners and occupational health service) in a large region in the Netherlands, the results are thought to be generalizable if the same kinds of treatment centres are available.

### Methodological considerations

A strength of the RCT design is the external validity, which is expected to be high due to the low comorbidity of the participants and uniform situational aspects of the study centre. However, contamination due to communication between participants cannot be ruled out completely as the study is conducted in one centre. An additional strength is the light condition in the CoPL group, which cannot be switched off completely. Only a small dose of non-effective light is used and this cannot be rated as effective or non-effective by the participant. The possibility exists that the combination with light therapy plus pulsed magnetic field therapy without coaching also is effective.

Another strength of the study is the use of hair cortisol analysis. Hair cortisol has the potential to fill the methodological gap of measuring chronic stress and is a unique, non-invasive means of capturing long-term cortisol secretion. Studies have shown that individuals who have elevated cortisol secretion due to stress demonstrate increased hair cortisol [23, 24]. As a biomarker of chronic stress, hair cortisol provides supplementary and objective insights beyond self-reported psychological levels of stress. Hair cortisol analysis can contribute to a more complete understanding of how long-term cortisol secretion mediates stress-related effects on health and well-being.

This study will provide information about the effectiveness of a new combined intervention of coaching with light therapy plus pulsed electromagnetic field therapy and on return to work, fatigue, stress, and quality of life. The results will be available in 2016. The intention is to implement the intervention in occupational care if it is shown to be effective.

## Abbreviations

Klx, Kilolux; lx, Lux; PEMF, Pulsed electro magnetic fields; RCT, Randomized controlled trial; SAD, Seasonal affective disorder; S-SASD, Subsyndromal seasonal affective disorder; UBOS, Utrecht burnout scale
